# Fungal Reactive Oxygen Species Secreted by *Candida albicans* Induce Barrier Disruption and Cell Death in HaCaT Keratinocytes

**DOI:** 10.3390/jof12010038

**Published:** 2026-01-02

**Authors:** Jayshree Low Jit Sze, Xinyue Chen, Kanami Orihara, Susumu Kajiwara

**Affiliations:** School of Life Science and Technology, Institute of Science Tokyo, Yokohama 226-8501, Japan; low.j.bd93@m.isct.ac.jp (J.L.J.S.); chen.x.ee0a@m.isct.ac.jp (X.C.); orihara.k.fc01@m.isct.ac.jp (K.O.)

**Keywords:** *Candida albicans*, NADPH oxidase, ROS production, tight junction

## Abstract

*Candida albicans* is a pathogenic fungus that expresses a fungal NADPH oxidase known as *C. albicans* Cfl11, which produces reactive oxygen species (ROS). Secretion of these ROS triggers caspase 3–mediated cell death in hepatocytes, which was attenuated in a mutant with a disrupted *CaCFL11* gene (designated *Cacfl11Δ* mutant). Here, we compared the effects of the *C. albicans* wild-type strain and the *Cacfl11Δ* mutant. Our findings revealed that *C. albicans* reduces the viability of HaCaT keratinocytes in a contact-independent manner. Furthermore, exposure to *C. albicans* increased intracellular ROS production and caspase 3 activity in HaCaT keratinocytes. These changes were attenuated when HaCaT keratinocytes were exposed to the *Cacfl11Δ* mutant or when HaCaT keratinocytes were treated with the known antioxidant N-acetylcysteine. Furthermore, wild-type *C. albicans,* but not the *Cacfl11Δ* mutant, disrupted transepithelial electrical resistance and modulated the downregulation of the tight-junction genes occludin and junction adhesion molecule 1 in HaCaT keratinocytes. Collectively, these results show that fungal ROS secretion via *CaCFL11* is a potent virulence factor in mediating keratinocyte viability and barrier function.

## 1. Introduction

Fungal infections cause approximately three million deaths each year, with invasive candidiasis accounting for an estimated one million of these deaths [1]. However, *Candida* infections have long been overlooked because they are often regarded as diseases of immunocompromised individuals. During the COVID-19 pandemic, both immunosuppressive drugs [2] and the SARS-CoV-2 virus itself [3] made patients more susceptible to secondary infections with opportunistic fungal pathogens [2]. Furthermore, a number of studies have reported an increase in antifungal resistance among *Candida* species [4,5,6,7,8]. Hence, additional fungal research, particularly focusing on *Candida* species, is vital to improve our understanding of *Candida* infection for future advancements in human health.

Despite being a major threat to human health, *Candida* spp., especially *C. albicans*, are frequently isolated from healthy individuals, and they are also often transmitted from mother to child [9,10]. However, in immunocompromised individuals, *C. albicans* transitions to a hyphal form that exhibits enhanced tissue invasion and increased damage to host tissues. Considerable research has focused on *Candida* infections in the gut, vagina, and oral cavity. However, *Candida*-related skin infections have been overlooked, despite a growing number of clinical reports of *C. albicans* infections in patients with diabetic foot ulcers [11,12], burn wounds [6,13] and atopic dermatitis [14]. In severe cases, a cutaneous *Candida* infection can progress to gangrene, ultimately requiring surgical intervention such as debridement or amputation.

Skin infections involving *C. albicans* can lead to a thickening of the skin, hyperkeratosis, and erythema [15]. The natural structure of the skin enables infections to be established only through active penetration and hyphal secretions. Several studies have confirmed that formation of hyphae plays a critical role in damage to the epithelial barrier [16,17]. The prominence of *C. albicans* infections in diabetic wounds [18] and in patients undergoing anti–IL-17 treatment for psoriasis [19,20] have narrowed *Candida* skin infection research efforts towards skin immune cell responses against *C. albicans*. However, the immune functions of keratinocytes are also impaired [21,22,23]. Although keratinocytes are often considered limited to detecting fungi through pattern-recognition receptors and modulating immune cell responses [24,25,26,27], they also contribute to forming a robust physical barrier via the expression of tight-junction (TJ) proteins [28,29].

TJs are structures composed of several proteins expressed on the cell membrane that tightly connect neighboring cells [29]. TJ proteins maintain the apical barrier by forming thread-like structures known as TJ strands along the cells. These proteins also form complexes with the cytoplasmic actin-binding protein zona occludens (ZO), which binds to the actin cytoskeleton to provide support and maintain cell polarity [30]. Although several TJ proteins are found in the skin, claudin 1 (*CLDN1*), occludin (*OCLN*), and junction adherence molecule 1 (*JAM1*) are the most abundant in cell lines and clinical samples [31,32]. *Candida albicans* is armed with a plethora of secretory factors. However, transepithelial electrical resistance (TEER) measurements have shown that the deletion of one or a few hyphae-associated factors, such as secreted aspartyl protease (SAP) and candidalysin [33,34], cannot completely prevent barrier damage, but inhibiting hyphae formation can [33,34,35]. This observation suggests that several hyphae-associated virulence factors mediate TJ damage. These studies also suggest that other *C. albicans* virulence factors may also play a role.

In our previous studies, we showed that *C. albicans* secretes reactive oxygen species (ROS), the synthesis of which is mediated primarily by the NADPH oxidase–encoding gene *CFL11*, also known as *FRE8* in *C. albicans*. We reported that fungal ROS generated via *Ca*cfl11 protein enhances transglutaminase 2 (TG2) activity in human hepatocytes, ultimately upregulating caspase 3–mediated apoptosis [36,37]. Deletion of the *CaCFL11* gene does not impede hyphae formation but does attenuate apoptosis in hepatocytes [36]. Furthermore, the equivalent homologue gene in *C. glabrata*, *CgNOX1*, also triggers ROS accumulation and apoptosis in hepatocytes [37,38], suggesting that fungal ROS represent a vital virulence factor shared by *C. albicans* and *C. glabrata*.

Interestingly, a previous study reported that exogenous ROS disrupt TJs. Direct exposure to H_2_O_2_ downregulates OCLN expression [39], disrupts the localization of CLDN1, and dampens TEER readings [40]. These observations prompted us to investigate the effects of ROS produced through *C. albicans* Cfl11 on HaCaT keratinocytes. In this study, we demonstrated that fungal ROS production via the *CaCFL11* gene contributes to the loss of barrier function and an increase in cell death. Furthermore, the reduction in keratinocyte viability, increase in caspase 3–mediated apoptosis, and downregulation of TJ protein genes were found to be contact-independent, thus highlighting the importance of *CaCFL11–*mediated ROS production in modulating skin keratinocyte viability and barrier function.

## 2. Materials and Methods

### 2.1. Culture of HaCaT Keratinocytes

Human HaCaT keratinocytes (CLS Cell Line, Heidelberg, Germany) were grown in Dulbecco’s Modified Eagle Medium (DMEM) (Fujifilm Wako Pure Chemical Corp., Osaka, Japan) supplemented with 10% fetal bovine serum (FBS; HyClone Cytivia, Tokyo, Japan) and 1% PenStrep (Gibco, Grand Island, NY, USA). The keratinocytes were serum starved for 24 h and then treated with 100 nM dexamethasone (Nacalai Tesque, Kyoto, Japan) for 2 h before infection. For indirect infection, yeast cells and HaCaT keratinocytes were separated using a 0.4-μm PET insert cup (Corning, Corning, NY, USA). For hydrogen peroxide (H_2_O_2_; Fujifilm Wako Pure Chemical Corp.) exposure, 800 μM H_2_O_2_ was added to DMEM + 10% FBS fresh before exposure of HaCaT keratinocytes. Medium supplemented with 5 mM *N*-acetyl-L-cysteine (NAC) (Sigma-Aldrich, St. Louis, MO, USA, CAS no. 616-91-1) was also prepared fresh before exposure of HaCaT keratinocytes.

### 2.2. Fungal Culture

All fungal strains used in this study are listed in Table 1. Fungi were pre-cultured in yeast peptone dextrose (YPD) medium at 30 °C with shaking. To prepare *C. albicans* for infection, the YPD medium was removed, and the fungal cells were washed with phosphate-buffered saline (PBS; Nacalai Tesque) and then resuspended in DMEM supplemented with FBS.

### 2.3. MTT([3-[4,5-Dimethylthiazol-2-yl]-2,5-Diphenyltetrazolium Bromide] Assay

MTT solution (5 mg/mL; Nacalai Tesque) was prepared fresh before the experiment. A total of 2 × 10^5^ HaCaT keratinocytes were seeded into 24-well plates and then indirectly exposed to 4 × 10^5^ fungal cells via an insert cup. At 24 h after indirect infection, MTT solution was added to each well at 10% of the culture volume and the cells were incubated at 37 °C for 2 h to allow for the formation of purple formazan. Medium containing MTT was then discarded, and the resulting formazan crystals were dissolved in dimethyl sulfoxide. The plates were then scanned using a Varioskan Lux (Thermo Fischer Scientific, Waltham, MA, USA) microplate reader at wavelengths of 670 nm and 560 nm. The formula used to calculate the percentage of viable keratinocytes is shown below. All groups were compared to the control, which was set at 100%.

(670 nm − 560 nm) × 100%

### 2.4. Lactate Dehydrogenase (LDH) Assay

LDH assays were performed using a Dojindo LDH assay kit (Dojindo Molecular Technologies, Inc., Kumamoto, Japan). Briefly, 1 × 10^1^ HaCaT keratinocytes were seeded in 96-well plates. At 24 h after direct infection with 2 × 10^1^ fungal cells, the LDH assay was performed, and the plates were scanned at 490 nm using a Varioskan Lux microplate reader. Fungus-only controls were also examined and showed readings similar to blank (medium only) samples.

### 2.5. Caspase 3 Activity

To measure caspase 3 activity, 4 × 10^5^ HaCaT keratinocytes were seeded onto round cover slips plated in a 12-well plate. After serum starvation and dexamethasone treatment, the keratinocytes were infected for 24 h either directly with 8 × 10^2^ fungal cells or indirectly with 8 × 10^5^ fungal cells. The cells were then fixed with 4% paraformaldehyde, blocked, and stained with a primary antibody against cleaved caspase 3 (Asp175) (1:400, Cell Signaling Technology, Danvers, MA, USA). The keratinocytes were then washed with PBS twice before staining with secondary antibody, anti-rabbit Alexa Fluor 488 (1:200, Jackson ImmunoResearch Laboratories, Inc. West Grove, PA, USA), and DAPI dye (1:5000) (Dojindo Molecular Technologies, Inc.). The keratinocytes were then analyzed using an LSM 780 laser scanning confocal microscope (Carl Zeiss, Inc., Jena, Germany). Representative images were acquired from at least 3 fields from 4 independent experiments. Fluorescence intensity associated with Alexa Fluor 488 was quantitated using ImageJ software (1.54g version).

### 2.6. Direct and Indirect Co-Culture of Fungi and HaCaT Keratinocytes

**Figure 1:**
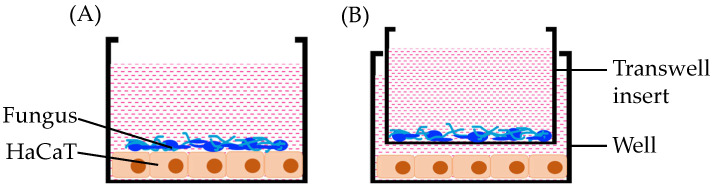
Direct (**A**) and indirect (**B**) infection models used in this study. Under both conditions, we confirmed that *Candida albicans* causes HaCaT keratinocyte death, whereas infection with non-pathogenic *Saccharomyces cerevisiae* did not alter HaCaT keratinocyte viability.

The effects of secreted fungal ROS on HaCaT keratinocytes were evaluated using two models (direct infection and indirect infection) established as shown in Figure 1. For direct infection, HaCaT keratinocytes were infected with fungal cells at a concentration equivalent to a multiplicity of infection (MOI) of 0.001. For the indirect infection model, HaCaT keratinocytes were exposed to fungal cells at a concentration equivalent to an MOI of 1.0, in which the HaCaT keratinocytes and fungal cells were separated via a PET 0.4-μm well insert. The infection ratio was determined by ensuring that neither infection with non-pathogenic *Saccharomyces cerevisiae* nor fungi alone affected cell viability readings, confirming that any changes observed were due only to the pathogenicity of *C. albicans*.

### 2.7. Measurement of Intracellular ROS Yield in HaCaT Keratinocytes During Fungal Co-Incubation

To measure intracellular ROS, 6 × 10^5^ HaCaT keratinocytes were seeded onto cover slips placed in 6-well inserts. After 48 h, the keratinocytes were infected for 24 h either directly with 1.2 × 10^3^ fungal cells or indirectly with 1.2 × 10^6^ fungal cells. To measure intracellular ROS, the medium was discarded, and a freshly prepared 5 μM CM-H_2_DCFDA solution was added. After incubation for 20 min at 37 °C with 5% CO_2_, the cover slips were washed once with PBS and then viewed under the ZEISS LSM 780 confocal microscope. Fluorescence was quantified using ImageJ software.

### 2.8. Determination of TEER

TEER was measured using a Millicell^®^ ERS-2 volt-ohm meter (Millipore, Burlington, MA, USA). A total of 1 × 10^4^ HaCaT keratinocytes were seeded in 0.4-μm PET insert cups and cultured in DMEM + 10% FBS + 2.4 mM calcium chloride for 6 days. The keratinocytes were then infected with 2 × 10^1^ yeast cells and suspended in DMEM + 10% FBS + 2.4 mM calcium chloride. TEER was measured before and after infection. Fungal cells alone were used as a negative control for direct infection and showed TEER values similar to the blank. All TEER values were normalized to the blank (medium only).

### 2.9. mRNA Collection and cDNA Synthesis

mRNA was extracted using Qiagen QiaZOL lysis buffer (Qiagen, Hilden, Germany), whereas cDNAs were synthesized using Toyobo REVTra-Ace reagent (Toyobo, Osaka, Japan) with a gDNA-remover kit. Briefly, HaCaT keratinocytes were washed with PBS, and then 500 μL of QiaZOL lysis buffer was added. Samples were collected, and mRNA was extracted following the supplier’s protocol. cDNA stocks of 50 ng/µL were prepared and stored at −80 °C.

### 2.10. Polymerase Chain Reaction (PCR) and Agarose Gel Electrophoresis Purification

All PCR experiments were performed using the Toyobo KOD FX Neo kit (Toyobo), following the manufacturer’s instructions. PCR products were purified using either the Qiagen QIAEX II kit (Qiagen) or the Promega Wizard SV Gel and PCR Clean-Up System (Promega Corporation, Madison, WI, USA). The final concentrations of PCR products were determined using 2% agarose gel electrophoresis and quantified with ImageJ software. Standards used for calibration were confirmed by sequencing on an Applied Biosystems 3730xl DNA Analyzer at the Integrative Bioscience Facility, Institute of Science, Tokyo, Japan.

### 2.11. Real-Time Quantitative Reverse Transcription PCR (qRT-PCR)

Absolute qRT-PCR assays were performed using a Toyobo SYBR Green kit (Toyobo) according to the manufacturer’s protocol, with a sample concentration of 5 ng/μL. All primers used in the study are listed in Table 2. Primers used in the study were human cell–specific and did not bind to fungal DNA, as confirmed by PCR. All data were normalized to 18S rRNA.

### 2.12. Statistical Analyses

All statistical analyses were performed using GraphPad Prism software (Prism 10). Quantitative data are shown as the mean ± standard deviation of at least three independent experiments. One-way analysis of variance was used to compare groups. All data shown are derived from three biological replicates, with at least two technical replicates. Differences were considered statistically significant at *p* < 0.05.

## 3. Results

### 3.1. Candida Albicans Reduces Cell Viability and Triggers Apoptosis in HaCaT Keratinocytes

We previously reported that *C. albicans* triggers caspase 3–mediated apoptosis in hepatocytes. In this study, we chose to expand our research findings to skin keratinocytes, as *C. albicans* may cause severe infections of the skin [18,19,20]. *C. albicans* infections of the skin are reportedly caused primarily as a result of hyphal attachment [15]. We constructed both direct and indirect *Candida* infection models to determine the fungal effect on HaCaT keratinocyte viability.

**Figure 2:**
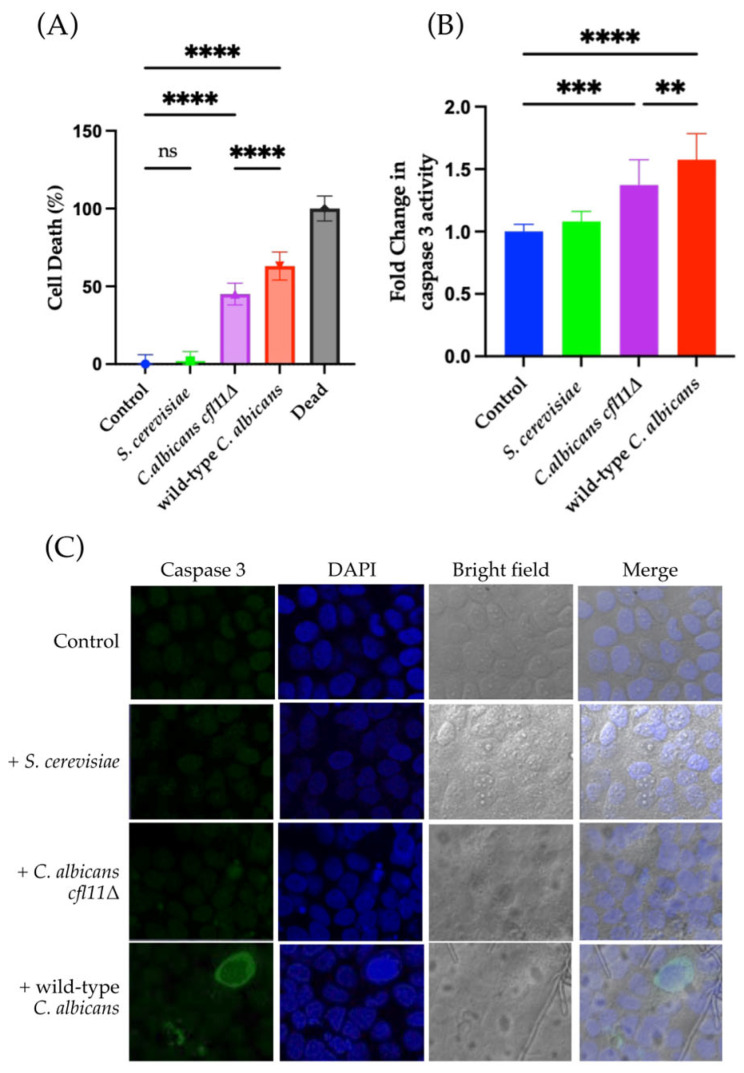
Direct infection with the *Candida albicans cfl11Δ* mutant (*C. albicans cfl11Δ*) resulted in reduced HaCaT keratinocyte death and caspase 3 activation. (**A**) HaCaT keratinocyte death upon direct infection with *Saccharomyces cerevisiae (S. cerevisiae),* the *C. albicans cfl11Δ* mutant, or the wild-type *Candida albicans (*wild-type *C. albicans)*, as measured by the LDH assay. Direct infection with the wild-type *C. albicans* induced approximately 63% keratinocyte death, whereas infection with the *C. albicans cfl11Δ* caused only 45% keratinocyte death. (**B**) Fold-change in caspase-3 activity following direct infection with *S. cerevisiae, C. albicans cfl11Δ* mutant, or the wild-type *C. albicans.* (**C**) Representative images of caspase 3 activity under each test condition. Direct infection with the wild-type *C. albicans* robustly activated caspase 3, and this activation was slightly attenuated in HaCaT keratinocytes infected with the *C. albicans cfl11Δ* mutant. Across all assays, infection with *S. cerevisiae* showed no significant changes and served as a negative control. All images shown are cropped. Full images shown in Figure S3. Data were analyzed using ordinary one-way analysis of variance with Tukey’s post hoc test. *p* < 0.0021 (**); *p* < 0.0002 (***); *p* < 0.0001 (****); no significant difference (ns).

As expected, direct infection with *C. albicans* triggered a high rate of keratinocyte death (Figure 2A and Figure S1A). By contrast, under our conditions, direct infection with a non-pathogenic yeast, *S. cerevisiae*, resulted in little to no keratinocyte death, thus confirming that the observed keratinocyte death was due only to the pathogenicity of *C. albicans.* In addition, a 1.5-fold increase in caspase 3 activity was detected, indicating the induction of apoptotic keratinocyte death (Figure 2B and Figure S1).

**Figure 3:**
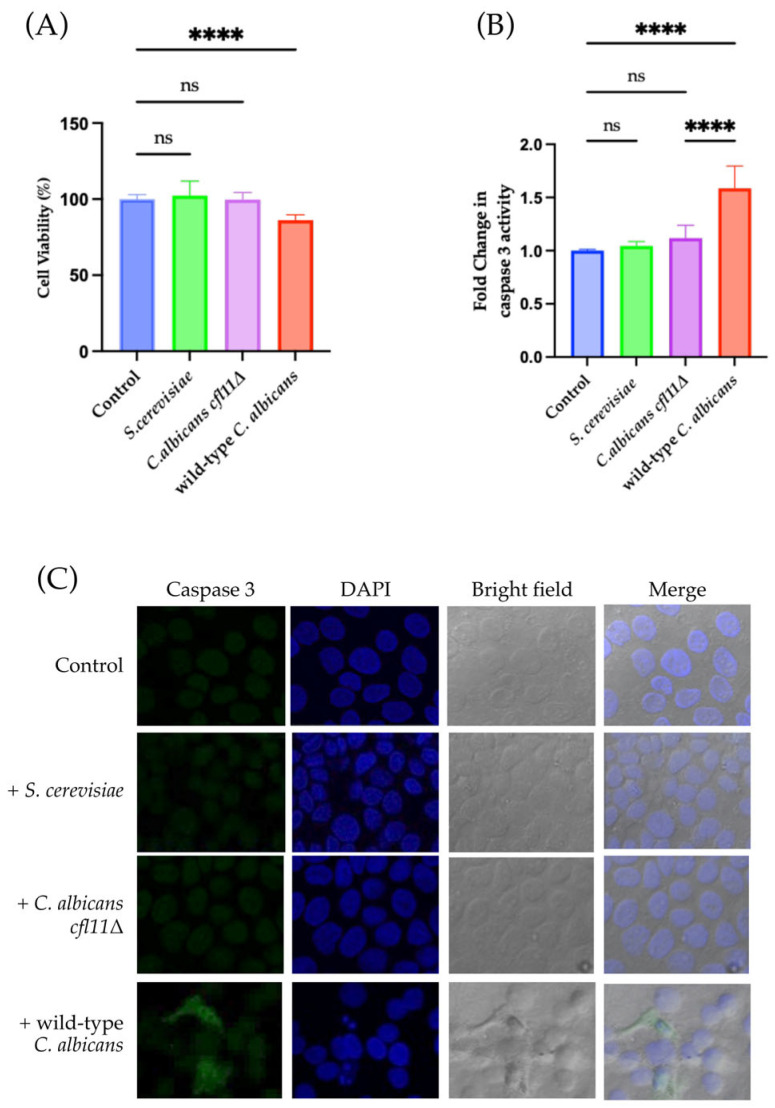
Indirect infection with the *Candida albicans cfl11Δ* mutant resulted in reduced HaCaT keratinocyte death and caspase 3 activation. (**A**) HaCaT keratinocyte death upon indirect infection with *Saccharomyces cerevisiae* (*S. cerevisiae*)*,* the *Candida albicans cfl11Δ* mutant (*C. albicans cfl11Δ*), or the wild-type *Candida albicans (*wild-type *C. albicans)* as measured by the MTT assay. Indirect infection with the wild-type *C. albicans* reduced keratinocyte viability by approximately 14%, whereas infection with the *C. albicans cfl11Δ* had no effect. (**B**) Fold-change in caspase 3 activity following indirect infection with *S. cerevisiae*, *C. albicans cfl11Δ*, or the wild-type *C. albicans.* (**C**) Representative images of caspase 3 activity under each test condition. Indirect infection with the wild-type *C. albicans* robustly activated caspase 3, and this activation was abrogated in keratinocytes infected with *C. albicans cfl11Δ.* Across all assays, infection with *S. cerevisiae* showed no significant changes and served as a negative control. All images shown are cropped. Full images shown in Figure S4. Data were analyzed using ordinary one-way analysis of variance with Tukey’s post hoc test. *p* < 0.0001 (****); no significant difference (ns).

Interestingly, indirect infection with *C. albicans* significantly decreased the viability of HaCaT keratinocytes by 14% (Figure 3A and Figure S2A), whereas viability was not affected by indirect exposure to *S. cerevisiae.* However, the pathogenicity was lower with indirect infection than direct infection, as expected. These results suggest that *C. albicans* affects not only hepatocytes [37] but also HaCaT keratinocytes. Furthermore, indirect infection with *C. albicans* also significantly increased caspase 3 activity by 1.5-fold (Figure 3B and Figure S2B), strongly suggesting that fungal secretions alone can modulate HaCaT keratinocyte viability and enhance caspase 3–mediated apoptosis. In both infection models, *C. albicans* increased caspase 3 activity compared with *S. cerevisiae*, strongly suggesting that *C. albicans* secretions significantly affect HaCaT keratinocyte viability and enhance caspase 3–mediated apoptosis.

### 3.2. C. albicans cfl11Δ Is a Significant Factor in Induction of Apoptosis in HaCaT Keratinocytes

Previous studies indicated that ROS secreted by *C. albicans* function as a virulence factor against human hepatocytes [36,37,38]. Therefore, to clarify whether fungal ROS are related to the induction of apoptosis in HaCaT skin keratinocytes, a *C. albicans CFL11* null mutant (*Cacfl11Δ*) was used in further experiments. The *CaCFL11* gene encodes NADPH oxidase, a major enzyme involved in the production of ROS by *C. albicans.* The *Cacfl11Δ* mutant shows reduced production of extracellular fungal ROS [36]. In the direct infection model, *Cacfl11Δ* induced a lower percentage of HaCaT keratinocyte death (45%) than did wild-type *C. albicans* (63%) (Figure 2A). The caspase 3 activity of HaCaT keratinocytes was also significantly lower in the mutant compared with the wild type (Figure 2B,C). By contrast, *Cacfl11Δ* did not affect the viability of HaCaT keratinocytes in the indirect infection model (Figure 3A). Moreover, *Cacfl11Δ* had a minimal effect on the caspase 3 activity of HaCaT keratinocytes in the indirect infection model (Figure 3B,C). These results suggest that *CFL11-*mediated ROS production in *C. albicans* is a potent trigger of human keratinocyte apoptosis.

**Figure 4:**
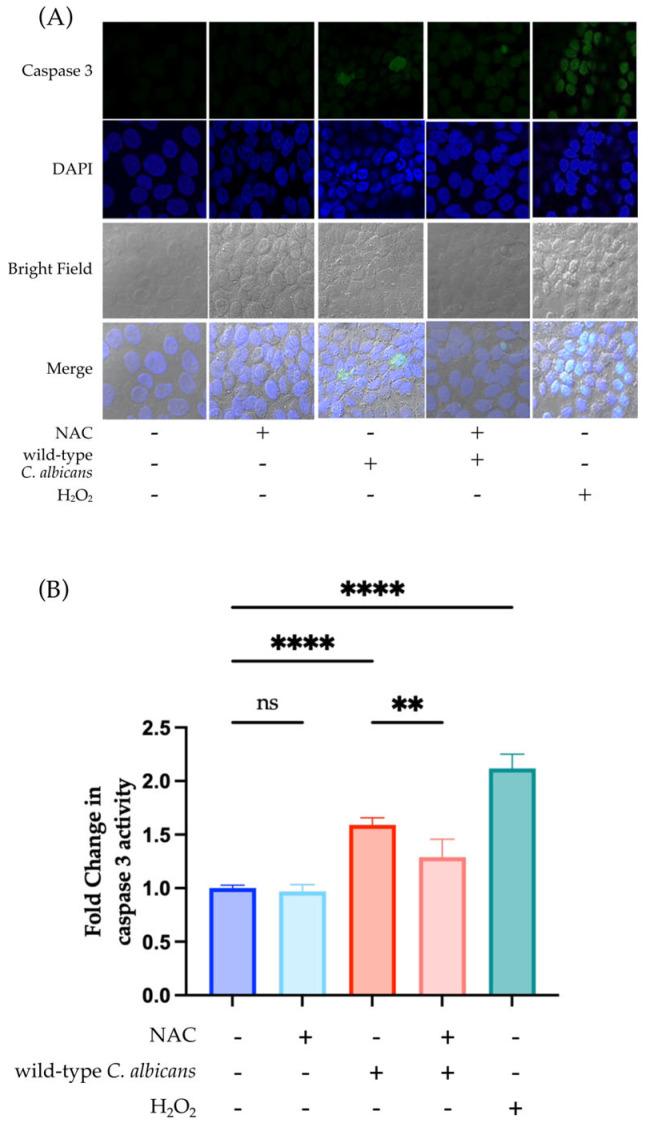
Exposure to 5 mM NAC alleviated the effect of *Candida albicans* (*C. albicans*) on caspase 3 activity in HaCaT keratinocytes. HaCaT keratinocytes were pretreated with 5 mM of the antioxidant NAC, before indirect exposure to the wild-type *C. albicans*. Immunostaining for cleaved caspase 3 was performed 24 h after co-incubation. (**A**) Representative images showing caspase 3 activity under each test condition. (**B**) Fold-change in caspase 3 activity following indirect infection with the wild-type *C. albicans*, NAC treatment, and exposure to H_2_O_2_ (positive control). Indirect infection with the wild-type *C. albicans* increased caspase-3 activity, whereas pretreatment with 5 mM NAC significantly reduced this activation. Exposure to a sublethal dose of H_2_O_2_ also triggered caspase 3 activity as a positive control. All images shown are cropped. Full images shown in Figure S5. Data were analyzed using ordinary one-way analysis of variance with Tukey’s post hoc test. *p* < 0.0021 (**); *p* < 0.0001 (****); no significant difference (ns).

To further confirm that fungal ROS leads to enhanced apoptosis of keratinocytes, HaCaT keratinocytes were treated with the antioxidant NAC in the indirect infection model to negate the fungal ROS effect during co-incubation with wild-type *C. albicans*. Although *C. albicans* upregulated the caspase 3 activity of HaCaT keratinocytes, NAC treatment significantly reduced—but did not completely abrogate—the caspase 3 activity (Figure 4). Furthermore, the addition of H_2_O_2_ also upregulated caspase 3 activity (Figure 4). This finding suggests that cell death and apoptosis of keratinocytes are affected by fungal ROS.

### 3.3. Fungal ROS Increase Intracellular ROS Production in HaCaT Keratinocytes

ROS secretion by *C. albicans* reportedly augments intracellular ROS production in hepatocytes, ultimately leading to caspase 3–mediated apoptosis of these cells [36]. Indeed, H_2_O_2_ reportedly has the same effect [44,45,46]. In HaCaT keratinocytes, H_2_O_2_ triggers accumulation of intracellular ROS which enhances the p53/Bax/Bcl-2–dependent pathway, upregulating p-53 and Bax expression while downregulating that of Bcl-2 [46]. This leads to the accumulation of cytochrome C, which activates caspase 9 and caspase 3 via apoptotic protease activating factor-1 [46,47].

**Figure 5:**
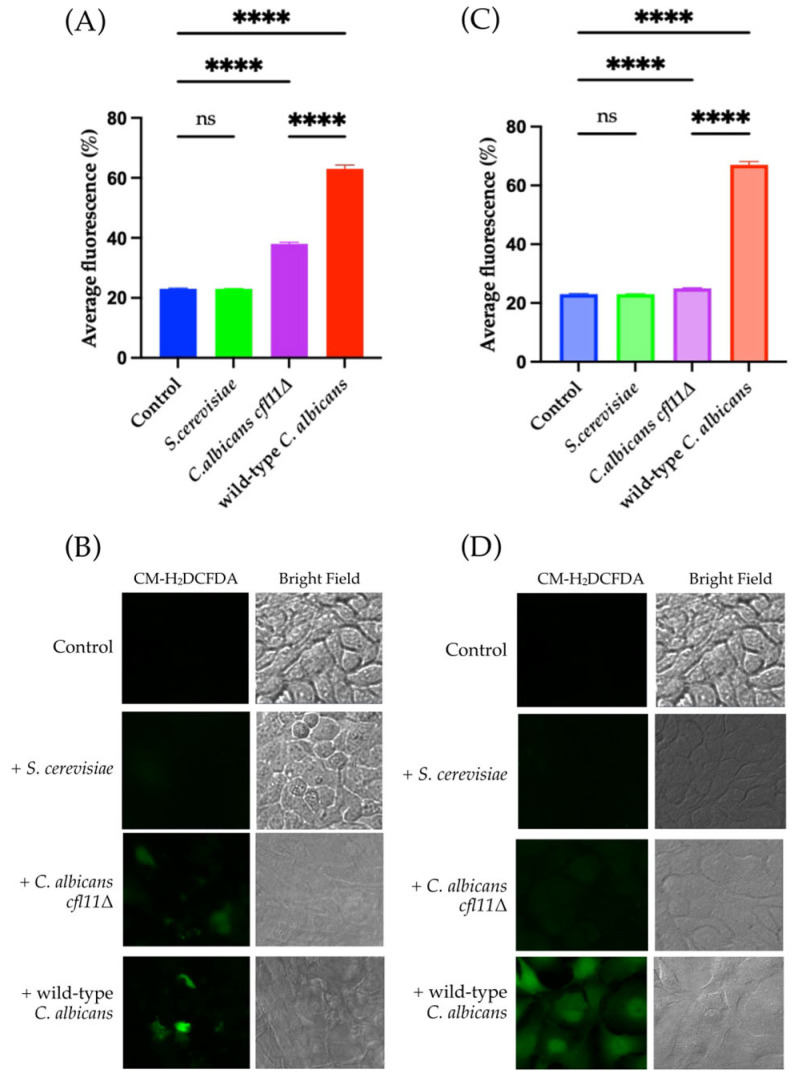
Intracellular ROS accumulation in HaCaT keratinocytes upon direct and indirect fungal infection. (**A**) Quantification of ROS levels (%) in HaCaT keratinocytes in the direct-infection model. (**B**) Intracellular ROS production in HaCaT keratinocytes was measured using 5 μM of CM-H2DCFDA following direct infection with the wild-type *Candida albicans* (wild-type *C. albicans*), the *Candida albicans cfl11Δ* mutant (*C. albicans cfl11Δ*), or *Saccharomyces cerevisiae* (*S. cerevisiae*). (**C**) Quantification of ROS levels (%) in HaCaT keratinocytes in the indirect-infection model. (**D**) ROS production in HaCaT keratinocytes upon indirect infection with the wild-type *C. albicans*, *C. albicans cfl11Δ*, or *S. cerevisiae*. All images shown are cropped. Full images shown in Figure S6. Data were analyzed using ordinary one-way analysis of variance with Tukey’s post hoc test. *p* < 0.0001 (****); no significant difference (ns).

Considering this previous literature, ROS accumulation in HaCaT keratinocytes during co-incubation with fungi was measured using the direct and indirect infection models. Upon direct infection with wild-type *C. albicans* and the *Cacfl11Δ* mutant, increased accumulation of intracellular ROS was observed in HaCaT keratinocytes (Figure 5A,B). By contrast, indirect exposure to the *Cacfl11Δ* mutant did not affect the intracellular ROS level in HaCaT keratinocytes (Figure 5C,D). Indirect exposure to wild-type *C. albicans* however, significantly increased intracellular ROS accumulation in HaCaT keratinocytes.

### 3.4. Fungal ROS Disrupt the Functional Barrier and Downregulate the Expression of TJ Gene mRNAs

**Figure 6:**
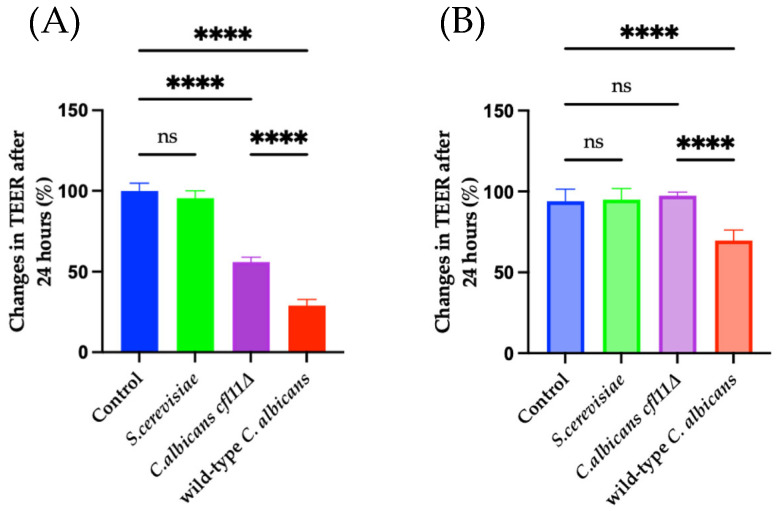
Changes in HaCaT barrier permeability upon infection with *Saccharomyces cerevisiae* (*S. cerevisiae*), *Candida albicans cfl11Δ mutant* (*C. albicans cfl11Δ*), or the wild-type *Candida albicans* (wild-type *C. albicans*). A decrease in TEER reflects increased barrier disruption. (**A**) In the direct-infection model, the wild-type *C. albicans* caused a marked decrease in TEER, indicating substantial barrier damage, whereas this effect was significantly—but not fully—attenuated in HaCaT keratinocytes infected with *C. albicans cfl11Δ* (**B**). In the indirect-infection model, the wild-type *C. albicans* induced a small but significant decrease in TEER, and this effect was completely abolished when using *C. albicans cfl11Δ*, suggesting that fungal ROS contributes to barrier disruption. *S. cerevisiae* did not alter TEER levels in either model. Overall, the TEER reductions observed were specific to *C. albicans* infection and were mitigated upon infection with the *C. albicans cfl11Δ* mutant. Data were analyzed using ordinary one-way analysis of variance with Tukey’s post hoc test. *p* < 0.0001 (****); no significant difference (ns).

The most important role of the skin is to shield the body from environmental insults. The expression of TJ proteins allows keratinocytes to bind tightly with neighboring keratinocytes, resulting in the formation of a signature “brick-and-mortar” structure [28,48]. It was previously reported that direct infection with *C. albicans* disrupts barrier strength in intestinal cells; however, whether *C. albicans* infection influences barrier function in the skin has not been reported. Furthermore, H_2_O_2_ has been reported to downregulate tight junction gene expressions [39] and protein arrangement [49]. Hence, the effects of *C. albicans* and fungal ROS on barrier-function disruption were analyzed in real-time in this study by monitoring TEER. Upon direct infection, both wild-type *C. albicans* and the *Cacfl11Δ* mutant significantly decreased the TEER of HaCaT keratinocytes, as shown in Figure 6A, although the effect of the *Cacfl11Δ* mutant was significantly attenuated. Furthermore, as shown in Figure 6B, indirect infection with wild-type *C. albicans* significantly reduced the TEER value by 30%, whereas indirect exposure to the *Cacfl11Δ* mutant had no observable effect on the TEER value of HaCaT keratinocytes. As expected, *S. cerevisiae* did not affect TEER values. These results strongly suggest that secretion of ROS by *C. albicans* plays a role in barrier disruption in HaCaT keratinocytes.

**Figure 7:**
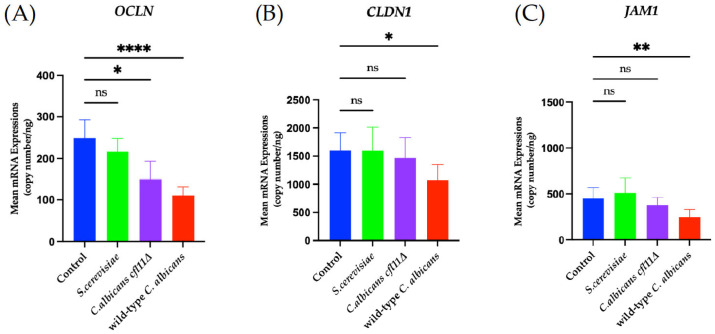
Changes in the mRNA expression of the tight junction-related genes *OCLN* (**A**), *CLDN1* (**B**), and *JAM1* (**C**) in HaCaT keratinocytes in the direct-infection model. HaCaT keratinocytes were directly infected with wild-type *Candida albicans (*wild-type *C. albicans*), the *Candida albicans cfl11Δ* mutant (*C. albicans cfl11Δ*) or *Saccharomyces cerevisiae (S. cerevisiae)*. Direct infection with the wild-type *C. albicans* significantly decreased the mRNA expression of *OCLN*, *CLDN1*, and *JAM1*. In contrast, infection with the *C. albicans cfl11Δ* attenuated these changes, with only *OCLN* showing a slight yet significant reduction. Infection with *S. cerevisiae* did not affect the mRNA expression of any of the tight junction-related genes. Data were analyzed using ordinary one-way analysis of variance with Tukey’s post hoc test. *p* < 0.0332 (*); *p* < 0.0021 (**); *p* < 0.0001 (****); no significant difference (ns).

**Figure 8:**
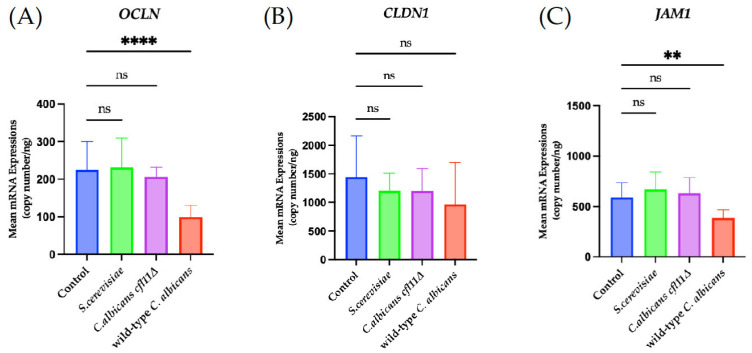
Changes in mRNA expression of the tight junction-related genes *OCLN* (**A**), *CLDN1* (**B**), and *JAM1* (**C**) in HaCaT keratinocytes in the indirect-infection model. HaCaT keratinocytes were indirectly infected with the wild-type *Candida albicans* (wild-type *C. albicans*), the *Candida albicans cfl11Δ* mutant (*C. albicans cfl11Δ*), or *Saccharomyces cerevisiae* (*S. cerevisiae*). Indirect infection with the wild-type *C. albicans* significantly decreased the mRNA expression of *OCLN* and *JAM1*, but not *CLDN1*. In contrast, infection with *C. albicans cfl11Δ* caused no significant changes in the mRNA expression of any tight junction-related genes. Indirect infection with *S. cerevisiae* also had no effect. Data were analyzed using ordinary one-way analysis of variance with Tukey’s post hoc test. *p* < 0.0021 (**); *p* < 0.0001 (****); no significant difference (ns).

To further characterize the effect of fungal ROS on the expression of TJ genes in skin keratinocytes, the mRNA expressions of *OCLN*, *CLDN1*, and *JAM1* was analyzed, as these three genes have been consistently reported across studies [31,43,50,51]. Direct infection of HaCaT keratinocytes with wild-type *C. albicans* caused a significant decrease in the mRNA expressions of all three TJ genes (Figure 7), whereas indirect exposure to the wild-type caused a significant decrease in *OCLN* (Figure 8A) and *JAM1* (Figure 8C). By contrast, direct infection of HaCaT keratinocytes with the *Cacfl11Δ* mutant led to a slight reduction in *OCLN* mRNA expression (Figure 7A), whereas indirect infection with the *Cacfl11Δ* mutant had no significant effect on the mRNA expression of all three TJ genes (Figure 8). We also confirmed that TJ gene expression was not affected by either direct infection or indirect exposure to *S. cerevisiae*.

**Figure 9:**
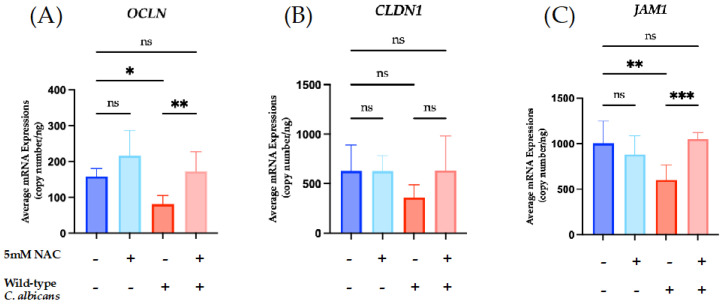
Changes in mRNA expression of the tight junction-related genes *OCLN* (**A**), *CLDN1* (**B**), and *JAM1* (**C**) in HaCaT keratinocytes in the indirect-infection model with 5 mM NAC treatment. HaCaT keratinocytes were indirectly infected with the wild-type *Candida albicans* in the absence or presence of 5 mM NAC. Indirect infection with wild-type *C. albicans* decreased the mRNA expression of *OCLN* (**A**) and *JAM1* (**C**), while *CLDN1* (**B**) remained unchanged. Treatment with 5 mM NAC and wild-type *C. albicans* significantly increased the mRNA expression of *OCLN* and *JAM1*, suggesting that quenching ROS improves the expression of tight junction-related genes in HaCaT keratinocytes. Data were analyzed using ordinary one-way analysis of variance with Tukey’s post hoc test. *p* < 0.0332 (*); *p* < 0.0021 (**); *p* < 0.0002 (***)**;** no significant difference (ns).

To further investigate whether the differences between wild-type *C. albicans* and the *Cacfl11Δ* mutant were caused by fungal ROS, HaCaT keratinocytes were treated with the antioxidant NAC and then indirectly infected with wild-type *C. albicans*. As shown in Figure 9, NAC treatment significantly alleviated the reduced mRNA expression of *OCLN* and *JAM1*, suggesting that secreted fungal ROS affect the mRNA expression of TJ protein genes.

## 4. Discussion

A number of factors that affect pathogenicity have been identified in *Candida albicans*, including morphological transition, adhesion, biofilm formation, secreted aspartyl proteases, and various secretory factors [15,52]. However, as fungal infection processes are complex, the molecular mechanisms underlying fungal pathogenicity are not completely clear. To better understand how *Candida*-derived ROS affect human keratinocytes, both direct and indirect infection models were used in this study to investigate the *C. albicans* infection mechanism. Whereas the direct infection model captured the effects of direct fungal interaction with human keratinocytes, the indirect infection model was used to observe the effects of fungal secretory factors, including fungal ROS. Under these conditions, we confirmed that a non-pathogenic yeast, *S. cerevisiae*, did not affect HaCaT keratinocyte viability in either model of infection.

When HaCaT keratinocytes were infected with wild-type *C. albicans* in both the direct and indirect infection models, a significant reduction in viability was observed. LDH assay results revealed that upon direct infection with wild-type *C. albicans*, keratinocyte viability declined to <50%. From this finding, we confirmed that direct infection with *C. albicans* exposes HaCaT keratinocytes to highly damaging effects associated with factors such as hyphal adhesion and inversion, in addition to the effects of secretory factors, ultimately resulting in a significant increase in the rate of keratinocyte death. However, indirect infection was shown to also reduce HaCaT keratinocyte viability significantly by 14%, suggesting that secretory factors play a significant role in keratinocyte death and viability. In addition, the caspase 3 activity in HaCaT keratinocytes was significantly increased by approximately 1.5-fold in both the direct and indirect infection models with wild-type *C. albicans*, suggesting that apoptosis plays an important role. Overall, our results indicate that these *C. albicans* secretory factors alone are capable of inducing apoptosis in keratinocytes.

In this study, the *Cacfl11Δ* mutant was used to investigate the effects of ROS secreted by *C. albicans* on HaCaT keratinocytes. The role of ROS as a pathogenic factor is further supported by the significant reduction in both keratinocyte death and caspase 3 activity upon direct and indirect infection with the *Cacfl11Δ* mutant. Moreover, to determine whether caspase 3 activity is enhanced by secreted fungal ROS, keratinocytes were treated with the antioxidant NAC to quench ROS secreted by *C. albicans*. NAC treatment significantly reduced caspase 3 activity upon indirect infection with wild-type *C. albicans*. This result suggested that fungal ROS trigger caspase 3–mediated death in HaCaT keratinocytes. Previous studies reported that increases in intracellular ROS lead to the activation of caspase 3–mediated apoptosis in skin keratinocytes [44,45,46,47]. In this study, we confirmed that ROS secreted via *C. albicans* Cfl11 induce increased accumulation of intracellular ROS in HaCaT keratinocytes. Hence, fungal ROS stimulate an increase in levels of intracellular ROS in HaCaT keratinocytes, thus activating caspase 3.

Our previous work showed that fungal ROS activate caspase 3–mediated apoptosis in hepatocytes by enhancing transglutaminase activity [37,53]. Transglutaminases play a vital role in the cornification [54] and inflammatory [55] processes in keratinocytes. The loss of transglutaminase 1 in mice is lethal due to a failure in proper cornified envelope formation; by contrast, the loss of transglutaminase 2 has no significant negative impact on the skin [56]. Furthermore, the inability of the current test of transglutaminase activity to differentiate between different transglutaminases underscores the need for knockout mutants to identify which if any of the transglutaminases are involved.

In addition to the induction of apoptosis of keratinocytes, we examined the effects of fungal ROS on TJ barrier function and the expression of TJ-related genes. In the skin, keratinocytes form a barrier that separates the inside and outside of the body; this barrier is formed through various TJ proteins that bind neighboring cells together, giving the skin its signature “brick-and-mortar” formation [28,48]. To measure TJ integrity and changes in barrier permeability, TEER assays were employed, as this approach permits continuous analysis of barrier permeability in the same samples. Wild-type *C. albicans* strongly impacted the barrier permeability of HaCaT keratinocytes in both the direct and indirect infection models. By contrast, *Cacfl11Δ* only increased the barrier permeability upon direct infection. Furthermore, in both models, *S. cerevisiae* had no effect on barrier function. Hence, although direct contact between *C. albicans* hyphae and host cells is important for barrier disruption, our study showed that factors secreted by *C. albicans*, including ROS, significantly affect the barrier function.

To determine the effects of *C. albicans* (including ROS secretion) on TJs at the transcription level, the expression of *OCLN*, *JAM1*, and *CLDN1* was analyzed, as these are the most widely reported TJ proteins expressed in skin cell lines and skin biopsies [31,57,58,59]. Direct infection with either the wild-type *C. albicans* or the *Cacfl11Δ* mutant significantly decreased the *OCLN* mRNA expression; however, the effect was less prominent upon infection with *Cacfl11Δ*. The downregulation in the mRNA expression of TJ-related genes was not observed upon direct infection with *S. cerevisiae* as a negative control. By contrast, unlike wild-type *C. albicans*, direct infection with the *Cacfl11Δ* mutant did not markedly affect the expression of *CLDN1* or *JAM1*. Furthermore, *OCLN* and *JAM1* were significantly downregulated upon indirect infection with wild-type *C. albicans* but not the *Cacfl11Δ* mutant. Although *CLDN1* appeared to be downregulated, the effects were not significant, suggesting that direct contact with *C. albicans* may be required to trigger a downregulation in *CLDN1* expression. Furthermore, our findings agree with the current literature; that is, disruption of one virulence factor alone is not sufficient to diminish the pathogenicity of *C. albicans*. However, future work targeting the *CaCFL11*, *CaECE1*, and *CaSAP* genes together could aid in developing ways to limit invasion, considering the effects of disruption or knockdown of single genes. Hence, investigations of double mutants may result in novel findings.

In conclusion, this study uncovered novel findings regarding the virulence of *C. albicans* against human skin keratinocytes. Although *C. albicans* can remain dormant on healthy skin, it becomes pathogenic in cases of weakened immunity, leading to severe skin barrier and tissue damage. We previously showed that ROS secreted by *C. albicans* trigger caspase 3–mediated cell death via transglutaminase 2. In this study, we report that fungal ROS not only trigger caspase 3–mediated apoptosis but also damage the barrier function of the skin and TJ formation at the transcription level. Considering the additional impact of fungal ROS and the different roles transglutaminases play in the skin, the results of this study may serve as a basis for future research to elucidate the molecular mechanism behind the effects of fungal ROS–mediated cell death and barrier disruption in human keratinocytes. In future studies, experiments using primary human keratinocytes are also needed to further strengthen our findings.

## Figures and Tables

**Table 1:** List of fungal strains.

Strain	Parent	Genotype	Reference
*S. cerevisiae* S288C		*MATα gal2 suc2 mal mel*	Kajiwara et al. 2000 [41]
*C. albicans* SC5314		Wild type	Fonzi & Irwin, 1993 [42]
*C. albicans cfl11Δ*	SC5314	*cfl11Δ::FRT*/*cfl11Δ::FRT*	Huang et al. 2020 [36]

**Table 2:** List of HaCaT cell primers used in this study.

Gene	Primer	Sequence	Reference
*18SrRNA*	18S-F	5′-CGC CGC TAG AGG TGA AAT TC-3′	This study
	18S-R	5′-CGA ACC TCC GAC TTT CGT TCT-3′	
*OCLN*	OCLN-F	5′-GCT TCA GTT GGT GTT GTG AG-3′	This study
	OCLN-R	5′-GAT GGC ATG GTG TAG TGT AG-3′	
*CLDN1*	CLDN1-F	5′-GGT GCT ATC TGT TCA GTG ATG-3′	This study
	CLDN1-R	5′-GGC TGA CTT TCC TTG TGT AG-3′	
*JAM1*	JAM1-F	5′-ACC TGG TTC AAA GAT GGG ATA G-3′	Leonardo et al. 2020 [43]
	JAM1-R	5′-TGT TGT GGG ATT CAG GAC ATA G-3′	

## Data Availability

The original contributions presented in this study are included in the article/supplementary material. Further inquiries can be directed to the corresponding author.

## Supplementary Materials

The following supporting information can be downloaded at: https://www.mdpi.com/article/10.3390/jof12010038/s1, Figure S1: Wild-type *Candida albicans* triggered HaCaT keratinocyte death and apoptosis of HaCaT keratinocytes upon direct infection; Figure S2: Wild-type *Candida albicans* reduced HaCaT keratinocyte viability and induced apoptosis of HaCaT keratinocytes upon indirect infection HaCaT keratinocytes were indirectly infected with wild-type *Candida albicans* (wild-type *C. albicans*) for 24 h before measurements were taken; Figure S3: Representative images of cleaved caspase 3 following direct infection; Figure S4: Representative images of cleaved caspase 3 following indirect infection; Figure S5: Representative images of cleaved caspase 3 following indirect infection and 5 mM NAC treatment; Figure S6: Representative images of HaCaT keratinocytes stained with 5 μM CM-H2DCFDA to measure intracellular ROS; Table S1: The efficiency of primers used in qRT-PCR assays.
