# Supplementary material for: Fungal Reactive Oxygen Species Secreted by Candida albicans Induce Barrier Disruption and Cell Death in HaCaT Keratinocytes
Source: J Fungi (Basel). 2026 Jan 2;12(1):38. doi: 10.3390/jof12010038 (PMC12842971; doi:10.3390/jof12010038)
Supplement: Supplementary file 1 [file jof-12-00038-s001.zip › jof-4032623-supplementary.pdf]

**Supplementary Data**

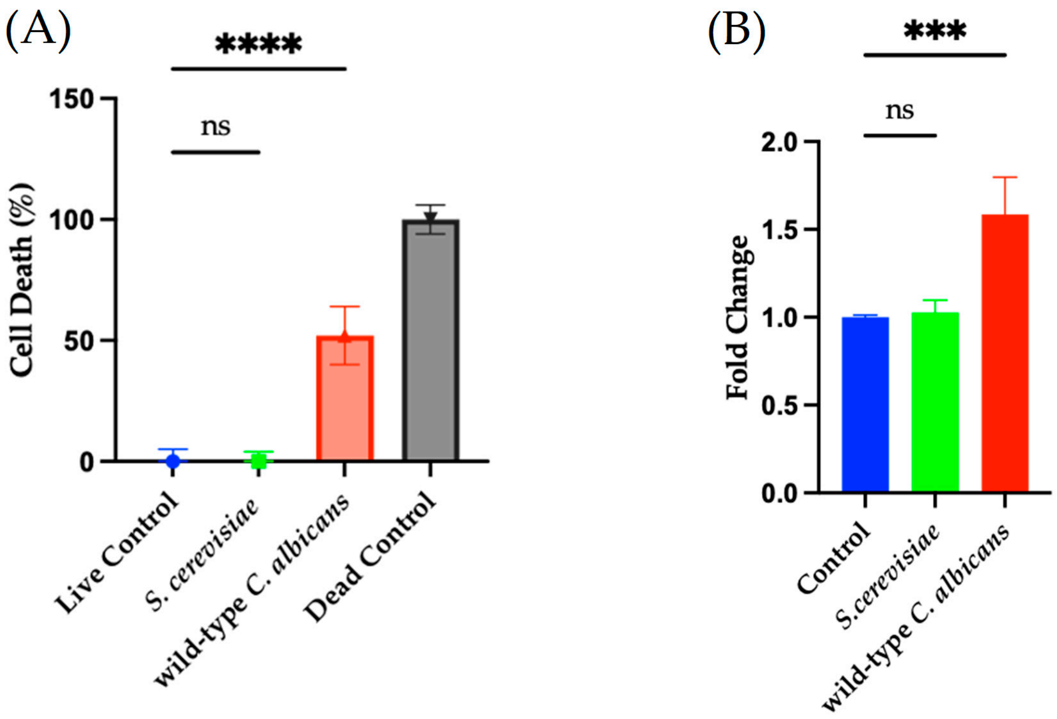


**Figure S1: Wild-type *Candida albicans* triggered HaCaT keratinocyte death and apoptosis of HaCaT keratinocytes upon direct infection.** *Candida albicans (C. albicans)* were directly seeded onto HaCaT keratinocytes for 24 h before measurements were taken. (A) Keratinocyte death upon direct infection was measured using the LDH assay. Infection with *Saccharomyces cerevisiae* (*S. cerevisiae*) alone showed negligible effects, while the wild-type *C. albicans* caused 52% death in HaCaT keratinocytes (B) Caspase 3 activity upon direct infection. While direct infection with *S. cerevisiae* showed no effect, the wild-type *C. albicans* showed a significant 1.5-fold increase in caspase 3 activity in HaCaT keratinocytes. All data were analyzed using ordinary one-way analysis of variance (ANOVA) with Tukey's post hoc test. $p$<0.0002 (***); $p$<0.0001 (****)

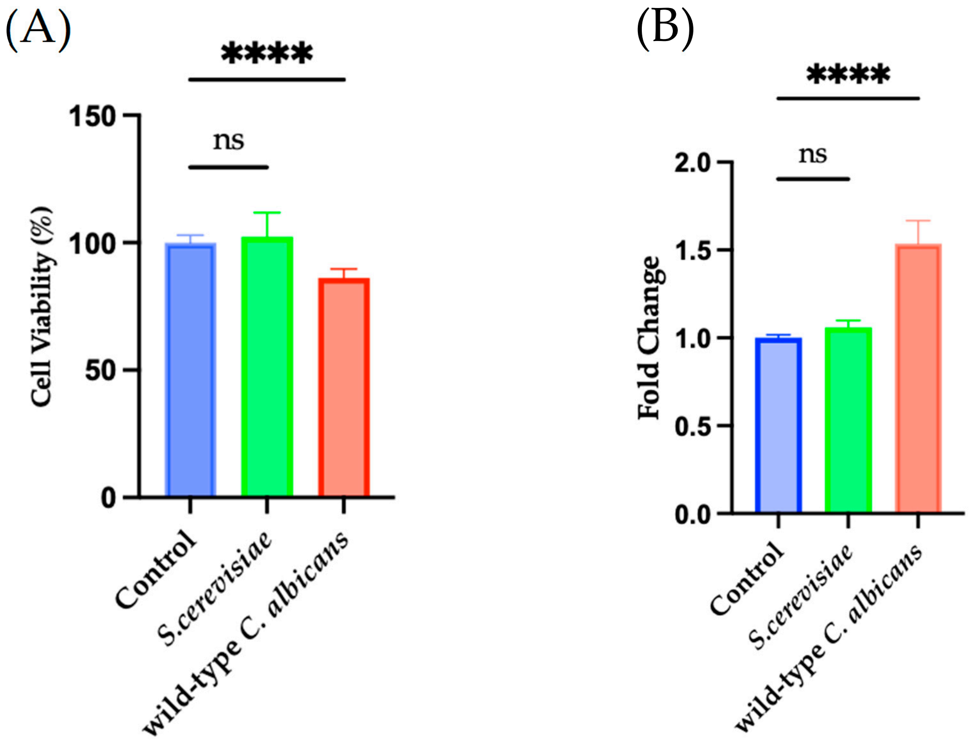


**Figure S2: Wild-type *Candida albicans* reduced HaCaT keratinocyte viability and induced apoptosis of HaCaT keratinocytes upon indirect infection** HaCaT keratinocytes were indirectly infected with wild-type *Candida albicans* (wild-type *C. albicans*) for 24 h before measurements were taken. (A) Keratinocyte death upon indirect infection was measured using the MTT assay. The indirect infection with *Saccharomyces cerevisiae* (*S. cerevisiae*) alone showed no effects, while the wild-type *C. albicans* reduced keratinocyte viability (B) Caspase 3 activity upon direct infection. While direct infection with *S. cerevisiae* showed no effect, the wild-type *C. albicans* showed a significant 1.5-fold increase in caspase 3 activity in HaCaT keratinocytes. All data were analyzed using ordinary one-way analysis of variance (ANOVA) with Tukey's post hoc test. $p$<0.0002 (***); $p$<0.0001 (****)

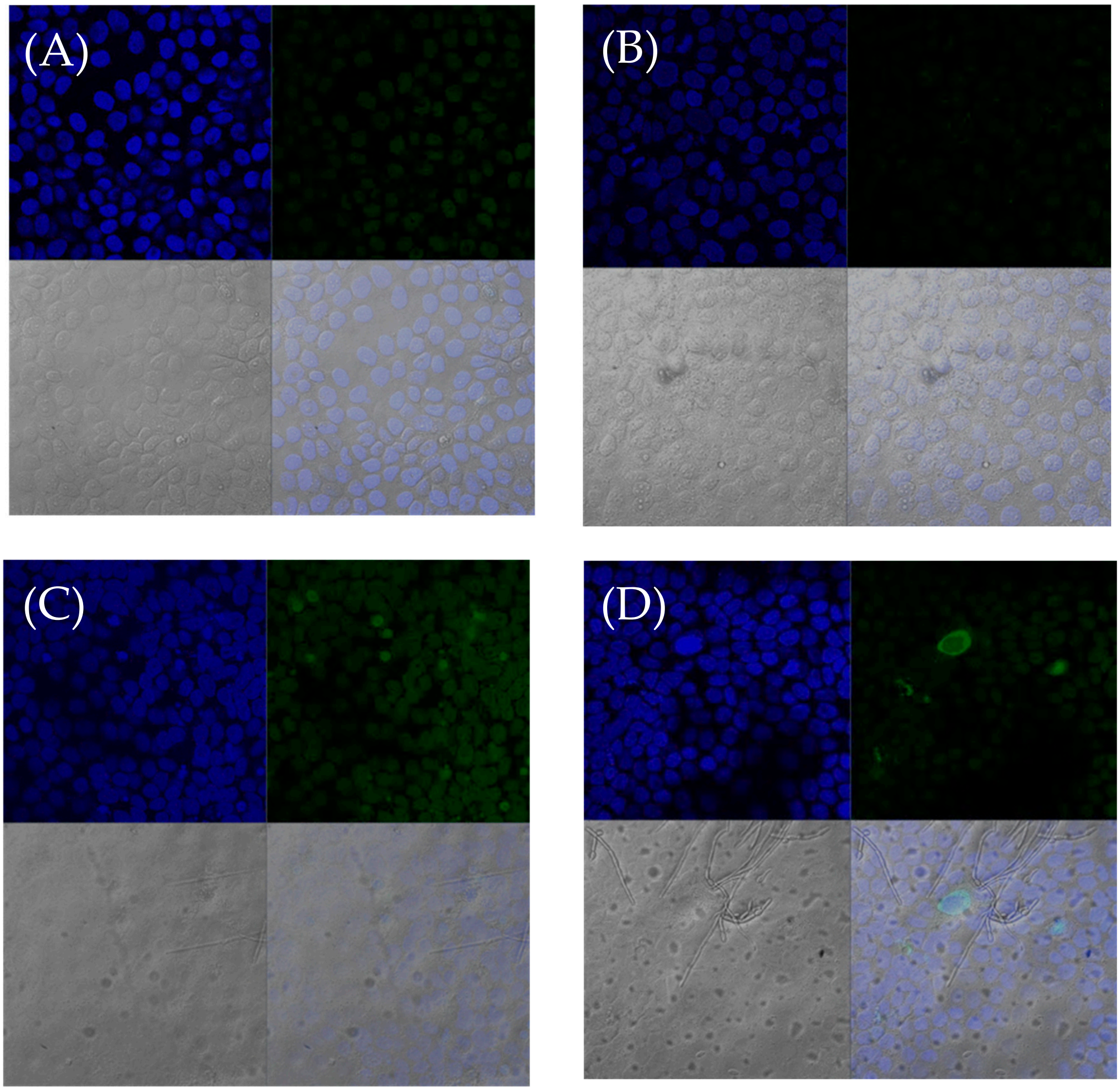


**Figure S3: Representative images of cleaved caspase 3 following direct infection.** The images shown in Figure 2C were cropped from these originals. Images were acquired at 40X magnification using oil immersion. (A) Control; (B) Infection with *Saccharomyces cerevisiae (S. cerevisiae)*; (C) Infection with *Candida albicans cfl11Δ* mutant (*C. albicans cfl11Δ*); (D) Infection with wild-type *Candida albicans* (wild-type *C. albicans*).

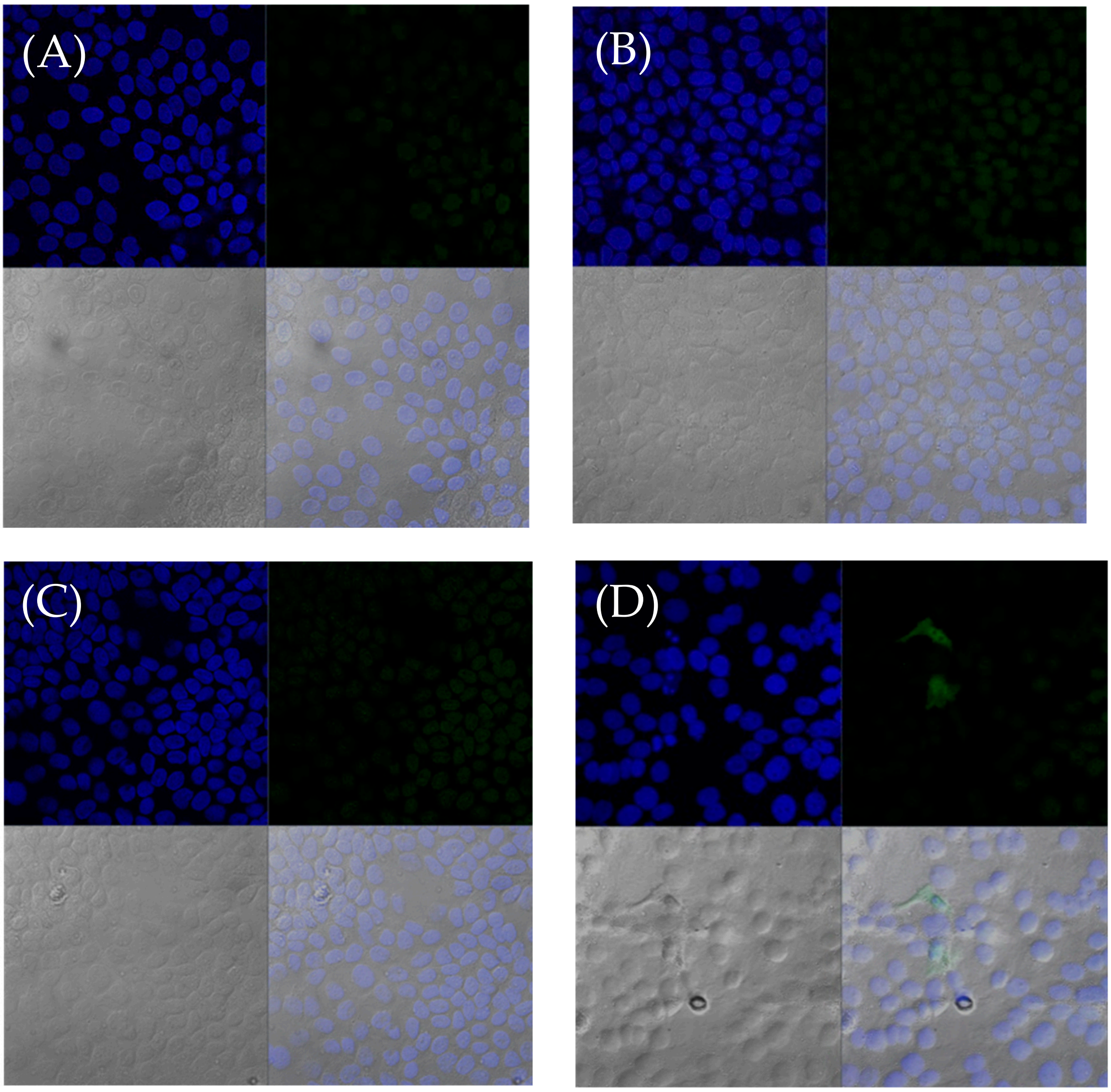


**Figure S4: Representative images of cleaved caspase-3 following indirect infection.** The images shown in Figure 3C were cropped from these originals. Images were acquired at 40× magnification using oil immersion. (A) Control; (B) Infection with *Saccharomyces cerevisiae (S. cerevisiae)*; (C) Infection with *Candida albicans cfl11Δ* mutant (*C. albicans cfl11Δ)*; (D) Infection with wild-type *Candida albicans* (wild-type *C. albicans*).

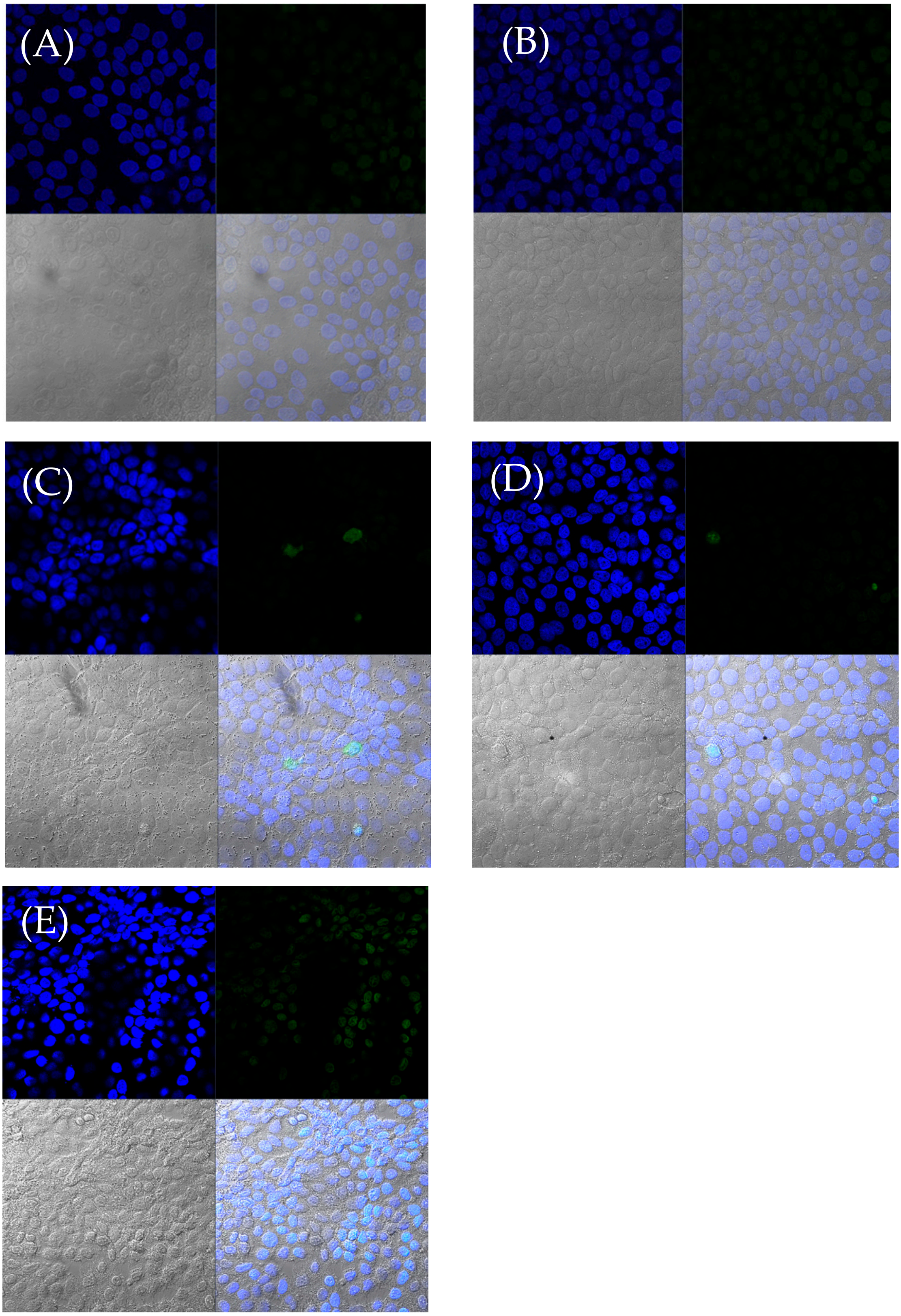


**Figure S5: Representative images of cleaved caspase-3 following indirect infection and 5mM NAC treatment.** The images shown in Figure 4A were cropped from these originals. Images were acquired at 40X magnification using oil immersion. (A) Control; (B) Control + 5mM NAC; (C) Infection with wild-type *Candida albicans* (wild-type *C. albicans*); (D) Infection with wild-type *C. albicans* + 5mM NAC (E) Keratinocytes treated with 800 µM of $H_2O_2$

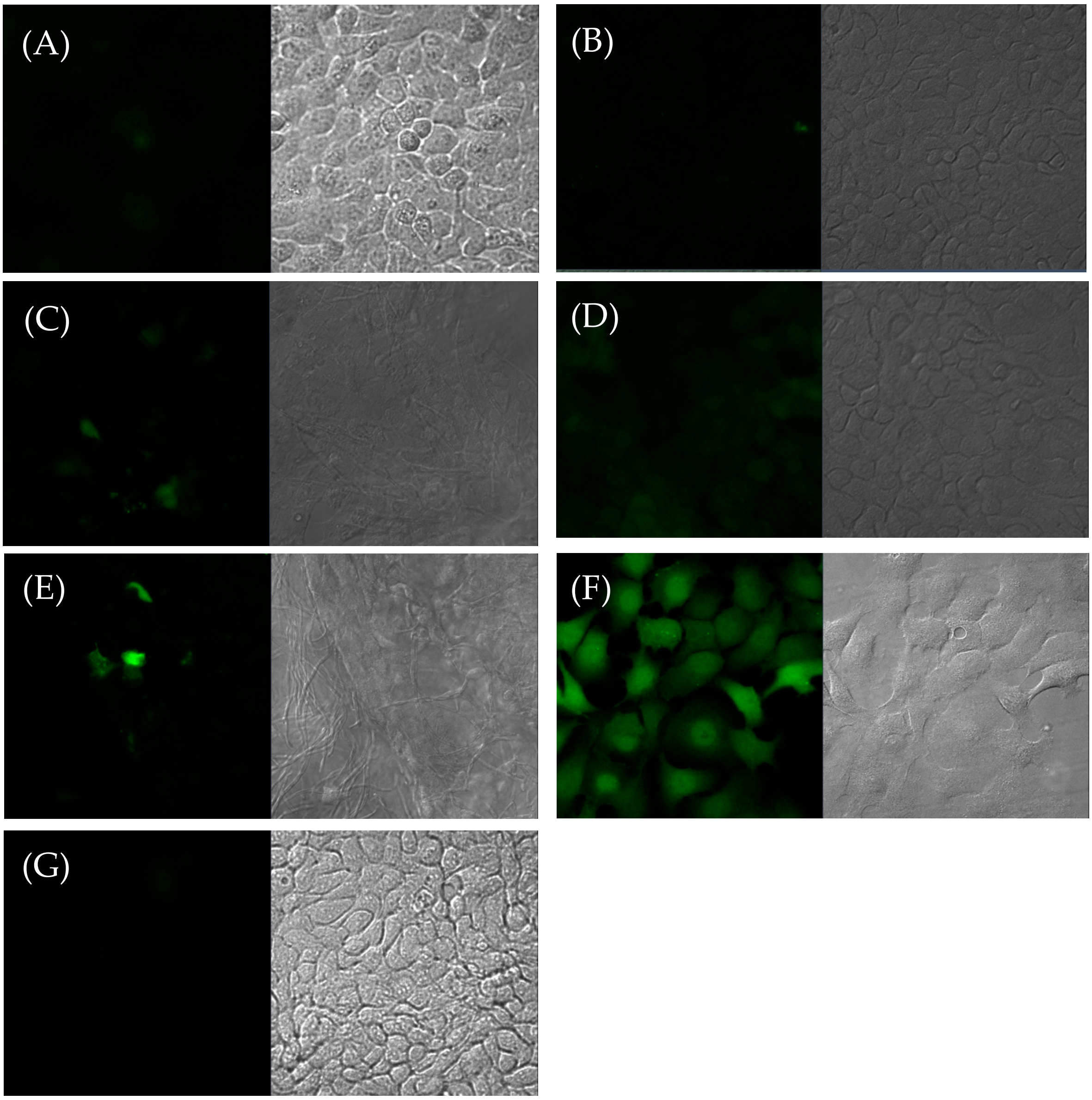


**Figure S6: Representative images of HaCaT keratinocytes stained with 5 μM CM-H2DCFDA to measure intracellular ROS.** The images shown in Figures 5A and 5C were cropped from these originals. Images were acquired at 40X magnification using oil immersion. The same control images were used. (A) Direct infection with *Saccharomyces cerevisiae (S. cerevisiae)*; (B) Indirect infection with *S. cerevisiae*; (C) Direct infection with the *Candida albicans cfl11Δ* mutant (*C. albicans cfl11Δ*); (D) Indirect infection with the *C. albicans cfl11Δ*; (E) Direct infection with the wild-type *Candida albicans (*wild-type *C. albicans)*; (F) Indirect infection with the wild-type *C. albicans*; (G) Control.

Supplementary Table S1: The efficiency of primers used in qRT-PCR assays

| Gene | Dilution Series | Standard curve slope | Intercept (Cq) | Efficiency (%) | $R^2$ | Melt peaks observed | Mean Tm of Amplicon ± SD (℃) |
|---|---|---|---|---|---|---|---|
| *18S* | $10^9$ - $10^3$ | -3.2718 | 36.27 | 2.02 | 1.00 | single | 82.23 - 82.80 |
| *OCLN* | $10^6$ - $10^0$ | -3.3320 | 33.12 | 2.00 | 1.00 | single | 81.61 - 81.97 |
| *CLDN1* | $10^6$ - $10^0$ | -3.3766 | 32.44 | 1.98 | 1.00 | single | 81.86 - 82.17 |
| *JAM1* | $10^6$ - $10^0$ | -3.3361 | 33.37 | 1.99 | 1.00 | single | 81.14 - 81.46 |
